# 16S rRNA metagenomic profiling of red amaranth grown organically with different composts and soils

**DOI:** 10.1007/s00253-023-12982-7

**Published:** 2024-01-15

**Authors:** Pooja Sharma, Sophayo Mahongnao, Arif Ahamad, Radhika Gupta, Anita Goel, Narendra Kumar, Sarita Nanda

**Affiliations:** 1 Department of Biochemistry, Daulat Ram College, University of Delhi, 4, Patel Marg, Maurice Nagar, Delhi, 110007 India; 2 Department of Environmental Science, Jamia Millia Islamia University, New Delhi, 110025 India

**Keywords:** Microbiome, 16S rRNA Metagenomics, Organic, Composts, Pathogens

## Abstract

**Abstract:**

In recent years organic food is gaining popularity as it is believed to promote better human health and improve soil sustainability, but there are apprehensions about pathogens in organic produces. This study was designed to understand the effect of different composts and soils on the status of the microbiome present in organically grown leafy vegetables. 16S rRNA metagenomic profiling of the leaves was done, and data were analyzed. It was found that by adding composts, the OTU of the microbiome in the organic produce was higher than in the conventional produce. The beneficial genera identified across the samples included plant growth promoters (*Achromobacter*, *Paenibacillus*, *Pseudomonas*, *Sphingobacterium*) and probiotics (*Lactobacillus*), which were higher in the organic produce. Some pathogenic genera, viz., plant pathogenic bacteria (*Cellvibrio*, *Georgenia*) and human pathogenic bacteria (*Corynebacterium*, *Acinetobacter*, *Streptococcus*, *Streptomyces*) were also found but with relatively low counts in the organic produce. Thus, the present study highlights that organic produce has lesser pathogen contamination than the conventional produce.

**Key points:**

*• 16S rRNA metagenomics profiling done for organic red amaranth cultivar*

*• Microbial richness varied with respect to the soil and compost type used*

*• The ratio of beneficial to pathogenic genera improves with the addition of compost*

**Graphical Abstract:**

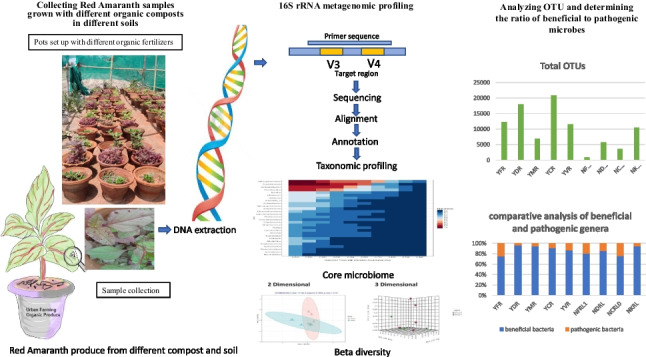

**Supplementary Information:**

The online version contains supplementary material available at 10.1007/s00253-023-12982-7.

## Introduction

Recently, the consumption of organic food has increased worldwide. Preference for organic food by consumers is attributed to their belief that organic produces are better than conventional produces. These beliefs are supported by studies which observed that organic produces have better nutritive value, such as higher levels of macronutrients (Worthington 2001), micronutrients (Hunter et al. 2011), vitamins-minerals (Leclerc et al. 2012), flavonoids (Gąstoł et al. 2011), beta-carotenoids (Hallmann et al. 2013), polyphenols (Faller and Fialho 2009), and lower levels of nitrates and nitrites (Hallmann et al. 2013;), potentially toxic elements (Baranski et al. 2014) and pesticide residues (Rekha et al. 2005), which promote health. Organic farming practices avoid chemical fertilizer, insecticides, and pesticides, making their production environmentally sustainable (Reganold and Wachter 2016). However, it is unclear whether organic products are contaminated with pathogenic microbes (Suciu et al. 2019). One of the studies reported that most organic and conventional vegetables possess similar microbial counts of mesophilic bacteria and total coliform (Kuan et al. 2017). Another study reported a significant difference in the microbial, molds, and *Escherichia coli* counts among the 189 organic and conventional produce, including leafy salads and fresh herbs (Becker et al. 2019). Conventional leafy vegetables generally have been reported to contain a higher microbial count of aerobic mesophilic bacteria, *E. coli*, and coliforms. However, detecting Enterobacteriaceae species in both systems suggested environmental contamination (Merlini et al. 2018). Another study observed that the farming system slightly affects the microbial composition of the leafy vegetables. They reported that *B. cereus*, *S. aureus*, and *L. monocytogenes*, which are associated with food-borne diseases, were high in organic produces due to the cross-contamination of organic produce from the adjacent conventional farm aided by environmental phenomena such as the storm and rain (Tango et al. 2014). Another study investigated the microbial contamination of leafy vegetables like kale, mustard, and spinach. They reported a significant difference with respect to the *E. coli* while no difference in total coliforms’ count (Shafie 2021). Whereas, the organic farm produce (certified) showed significantly higher microbial contamination of *E. coli* (Szczech et al. 2018). Therefore, it is believed that using animal waste manure and sewage sludge in organic farming could be a source of microbial contamination (Oliveira et al. 2010).

Thus, the above studies indicated that microbial contamination may or may not be dependent on the farming practice systems alone. To understand this, we formulated a hypothesis, “Organic produce has higher counts of both the beneficial and pathogenic microbes,” and designed this present study to compare and analyze the microbial composition of red amaranth grown organically, using different urban organic composts such as the leaf waste compost, municipal waste compost, kitchen waste compost, vermicompost, and cow dung manure. These were compared to the conventional produce (control) grown using diammonium phosphate (DAP). Two different soils, Yamuna flood plain soil and residential garden soil, were used in this study, to observe the effect of soil on the organic growth of cultivars.

## Materials and methods

### Leafy vegetables plantation and sample preparation

In the summer season plantation of each leafy vegetable was done with a particular type of fertilizing input (LWC, MWC, CDM, KWC, VC, and DAP) in the popular urban terrace farming setup. The organic composts were collected locally at Delhi-Nation Capital Region. The leaf waste compost was collected from DHARA Recycle Unit at the Daulat Ram College campus where leaf waste compost is produced by collecting and composting the leaf waste generated in the campus itself. The kitchen waste which is generally prepared by composting kitchen waste was collected from kitchen welfare association. The cow dung manure and vermicompost were collected from local farmers. The municipal waste compost was collected from Municipal Corporation of Delhi near Okhla landfill site, New Delhi, and the DAP fertilizer was obtained from a general store.

This experiment was done in two sets with soil from two different origins, viz., soil A, taken from river flood plains of Yamuna river basin of National Capital Territory of Delhi, and soil B, taken from residential soil of institutional garden of Daulat Ram College for women, University of Delhi. The seeds of leafy vegetables, viz., *Spinacia oleracea* (spinach), *Amaranthus viridis* (green amaranth), and *Amaranthus cruentus* (red amaranth) that belonged to the same family *Amaranthaceae*, were obtained from Krishi Vigyan Kendra, National Horticulture Research and Development Foundation, Ujwa, New Delhi.

### Potting

In the present study, the leafy vegetables were grown organically as well as conventionally in the same season, under same environmental conditions. The controls were the leafy vegetable grown conventionally using DAP (diammonium phosphate), calcium, and phosphate in a ratio of 4:1:1 in grams per kg of soil. The potting was done in the first week of April which had recorded the temperature of 29–40 °C with an average humidity of 34–44%; and harvesting was done in the late June. The potting was done according to a urban farming set up with soil and compost taken in a ratio of 1:0.25. The soil mixes, leaf waste compost soil mix (LWCS), municipal waste compost soil mix (MWCS), cow dung manure soil mix (CDMS), kitchen waste compost soil mix (KWCS), and vermicompost soil mix (VCS), were prepared in both the soil, viz., soil A and soil B with particular type of compost (LWC, MWC, CDM, KWC, and VC).

### Sample preparation

After harvesting, the leaves of different produce, viz., fertilizer produce (YFR), leaf waste compost produce (YDR), municipal waste compost produce (YMR), cow dung manure produce (YCR), and vermicompost produce (YVR) from soil A (group 5); and fertilizer produce (NFRL1), leaf waste compost produce (NDRL), cow dung manure produce (NCRL0), and kitchen waste compost produce (NKRL) from soil B (group 7) were collected and without any washing were packed in a zip-lock bags for further analysis (Achikanu et al. 2013).

### DNA extraction and PCR amplification of V3-V4 region of 16 s rRNA gene

DNA extraction was done using the suitable method for the sample type from commercially available kits such as Qiagen, Zymo Research, and Thermo-Fisher. DNA extraction was done as per the manufacturer’s recommendation. The extracted DNA from the samples was subjected to Nano Drop and GEL check before being taken for PCR amplification: The Nano Drop readings of 260/280 at a value of 1.8 to 2 were used to determine the DNA’s quality.

For the metagenomic analysis, the extracted DNA was amplified and sequenced to obtain the DNA sequence of the V3–V4 region of the 16S rRNA bacterial gene. The amplification was performed using a PCR mix containing: high-fidelity DNA polymerase, 0.5 mM dNTPs, 3.2 mM MGk_2_, and PCR enzyme buffer. The primers used were 16sF:-5′ AGAGTTTGATGMTGGCTCAG 3′ and 16sR:- 5′ TTACCGCGGCMGCSGGCAC 3′. The conditions for the PCR amplification were as follows: 40 ng of extracted DNA was used for amplification along with 10 ρM of each primer. The initial denaturation was set at 95 °C. The 25 cycles were set with the following condition: denaturation at 95 °C for 15 s, annealing at 60 °C for 15 s, elongation at 72 °C for 2 min, and final extension at 72 °C for 10 min and hold at 4 °C. The amplified 16 s PCR product is purified and subjected to gel check and Nanodrop quality control. The Nanodrop readings of 260/280 at a value of 1.8 to 2 were used to determine the DNA’s quality.

### Overview of sequencing protocol and bioinformatics protocol

The amplicons from each sample were purified with Ampure beads to remove unused primers, and additional eight cycles of PCR were performed using Illumina Miseq barcoded adapters to prepare the sequencing libraries. Libraries were purified using Ampure beads and quantitated using a Qubit dsDNA high sensitivity assay kit. Sequencing was performed using Illumina Miseq with 2 × 300PE v3 sequencing kit.

The raw data quality control (QC) was done using FASTQC and MULTIQC, followed by the trim of adapters and low-quality reads by TRIMGALORE. The trimmed reads were further taken for the process, which included merging paired end reads, chimera removal, and OTU abundance calculation and estimation correction; this was achieved by QIIME/MOTHUR/KRAKEN/BRACKEN workflows. This workflow enables highly accurate investigations at the genus level. The databases used were SILVA/GREENGENES/NCBI. Each read was classified based on % coverage and identity. The 16S workflow helps identify pathogens in a mixed sample and understand a microbial community’s composition.

The raw data obtained using Illumina Miseq was deposited at the INDA (Indian Nucleotide Database Archive) of the Indian Biological Database Centre (IBDC) with a reference INDA study/BioProject accession no. INRP000068. The International Nucleotide Sequence Database Collaboration (INSDC) accession numbers were generated for each sample. The sample codes along with their respective accession numbers are given as YFR-ERS15561320, YDR-ERS15561348, YMR-ERS15561353, YCR-ERS15561430, YVR-ERS15561350, NFR-ERS15561351, NDR-ERS15561352, NCR-ERS15561856, and NKR-ERS15561858.

## Results

### The number of reads, GC content, and total OTUs

The operational taxonomic units (OTU), generated from sequencing the V3–V4 region of the 16S rRNA gene, represented the individual microbial count and thus was a direct measure of the microbial richness. In general, the OTU was in the range of 10,000–20,000 in soil A produce, while soil B produce had much lesser OTU, in the range 800–10,000. Overall, the produce grown in soil A with cow dung manure had the highest number of reads, OTU, highest GC content of 55%, and had the highest library size indicating greatest microbial richness, followed by the leaf waste compost produce, which was at the second high level with respect to both the OTU number and the library size. Comparing the total OTUs of the conventional produce in both soils, it was observed that the total OTU of soil A fertilizer produce was much higher than the soil B fertilizer produce. The OTUs in both soils increase on addition of organic waste composts **(**Table 1).

**Table 1:** The number of reads, GC content, and the total number of OTUs (operational taxonomic units): this table shows the increment in the OTU among the compost produce compared to the fertilizer produce

Compost type	Number of reads (in millions)	GC content (%)	Total OTUs
Soil A fertilizer produce (YFR)	0.2	55	12,314
Soil A leaf waste compost produce (YDR)	0.2	53	17,960
Soil A municipal waste compost produce (YMR)	0.05	52.5	6886
Soil A cow dung manure produce (YCR)	0.2	55	20,866
Soil A vermicompost produce (YVR)	0.2	54.5	11,551
Soil B fertilizer produce (NFRL1)	0.09	53.51	875
Soil B leaf waste compost produce (NDRL)	0.07	53.5	5715
Soil B cow dung compost (NCRL0)	0.05	53.50	3586
Soil B kitchen waste compost produce (NKRL)	0.09	53	10,404

### Microbiome diversity

#### Alpha and beta diversity

**Fig. 1:**
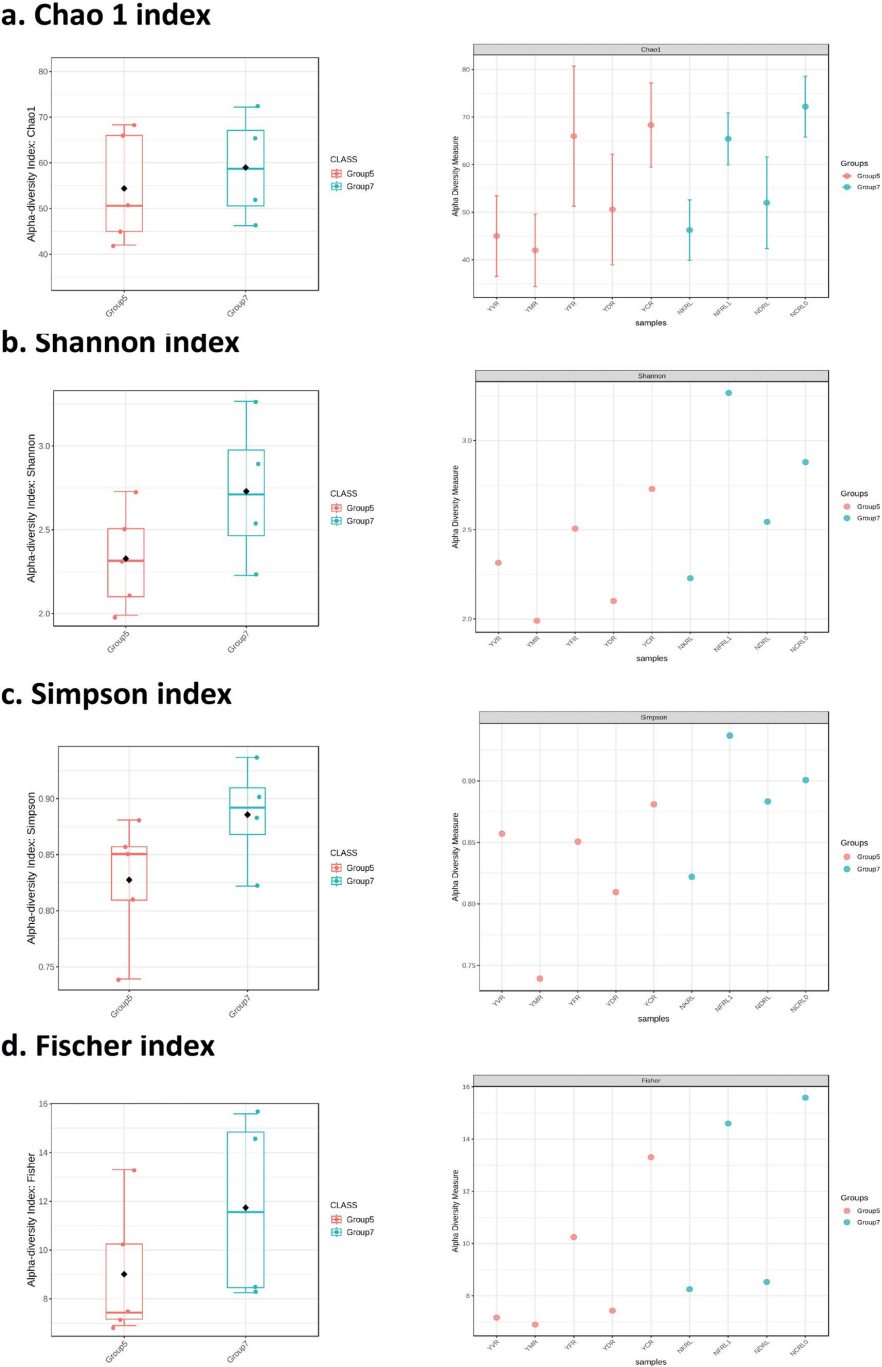
Alpha diversity analysis: this figure shows the alpha diversity index measured with four methods viz., Chao1, Shannon, Simpson, and Fisher

All samples were rarefied to even the sequencing depth based on the sample having lowest sequencing depth, and the analysis was visualized with the filtered data source. The alpha diversity was measured using four metrics, Chao1, Shannon, Simpson, and Fisher, with the statistical method of *T*-test/ANOVA. The Chao 1 index and Fisher index represents the species richness considering the species diversity. In contrast, Shannon and Simpson represent species richness and the evenness with which a species is distributed in a population. Simpson index gives more weightage to the species richness. The Chao 1 index computed for identifying the alpha diversity showed that the cow dung manure produces from both the soil types had the highest alpha diversity (soil A, Chao 1 index = 70; Shannon index = 2.71; Simpson index = 0.87; Fisher index = 13.3; soil B, Chao 1 index = 70.5; Shannon index = 2.90; Simpson index = 0.90; Fisher index = 15.6), while the municipal waste compost produce in soil A had the least alpha diversity and the most uneven species distribution (Chao 1 index = 39; Shannon index = 1.99; Simpson index = 0.75; Fisher index = 6.6). The *p*-value in Chao1, Shannon, Simpson, and Fisher alpha diversity index was measured to be 0.45803, 0.20568, 0.12833, and 0.81583 with the ANOVA *F*-value of − 0.78777, − 1.4653, − 1.732, and − 2.2041 (Fig. 1).

**Fig. 2:**
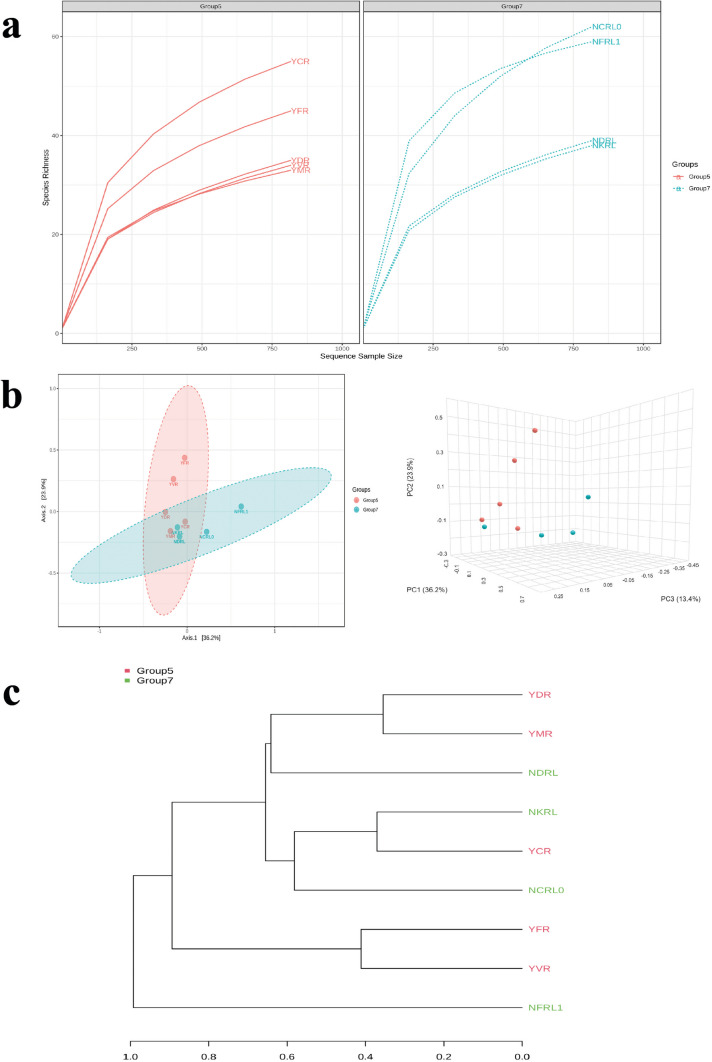
**A** Rarefaction curve of all the produce: the refraction curve shows that the cow dung manure produce in both the soil has highest species richness. **b** Beta diversity analysis: this figure shows the two-dimensional and three-dimensional beta diversity constructed at the taxonomic level of genus with the Bray–Curtis index distance method. **c** Dendrogram analysis of all the produce: the constructed dendrogram shows the ancestral homogeneity of the urban organic compost produce (YDR, soil A leaf waste compost produce; YMR, soil A municipal waste compost produce; NKRL, soil B kitchen waste compost produce)

The rarefaction curve of both the soil produces showed that the species richness followed the same order in both soils A and B. The cow dung manure and the fertilizer produce had the highest species richness. In contrast, the soil A produces with municipal and vermicompost had relatively lower species richness than the conventional produces. Similarly, in the case of the produces from soil B, cow dung manure produce and fertilizer produce had the highest species richness followed by the NDRL and NKRL (Fig. 2a).

The beta diversity measured the species richness between two communities and was constructed at the taxonomic level of genus with the Bray–Curtis index distance method based on permutational MANOVA (PERMANOVA) statistical method with *p* < 0.127 and *f*-value of 1.7128. It was observed that YDR, YMR, YCR, NDRL1, and NKRL, the group of urban organic waste compost produces, clustered together. Also, the samples, viz., NCRL0 and YVR, were placed very close to this cluster group. While the conventional produce, YFR and NFR, of both the soils was placed distant from this cluster group. The dendrogram prepared on the Bray–Curtis index with the Ward clustering algorithm showed that the groups YDR and YMR were closely related but substantially different from the other groups, YCR, YFR, and YVR. Similarly, the groups NFRL1 and NCRL0 were closely related but substantially different from the other groups, NDRL and NKRL. NDRL was different from the other groups like the NFRL1 and NCRL0 but substantially close to other groups, YDR, YMR, NKRL, and YCR (Fig. 2b, c).

### Taxonomic classification and identification of beneficial and pathogenic microbes

#### Phylum level

**Fig. 3:**
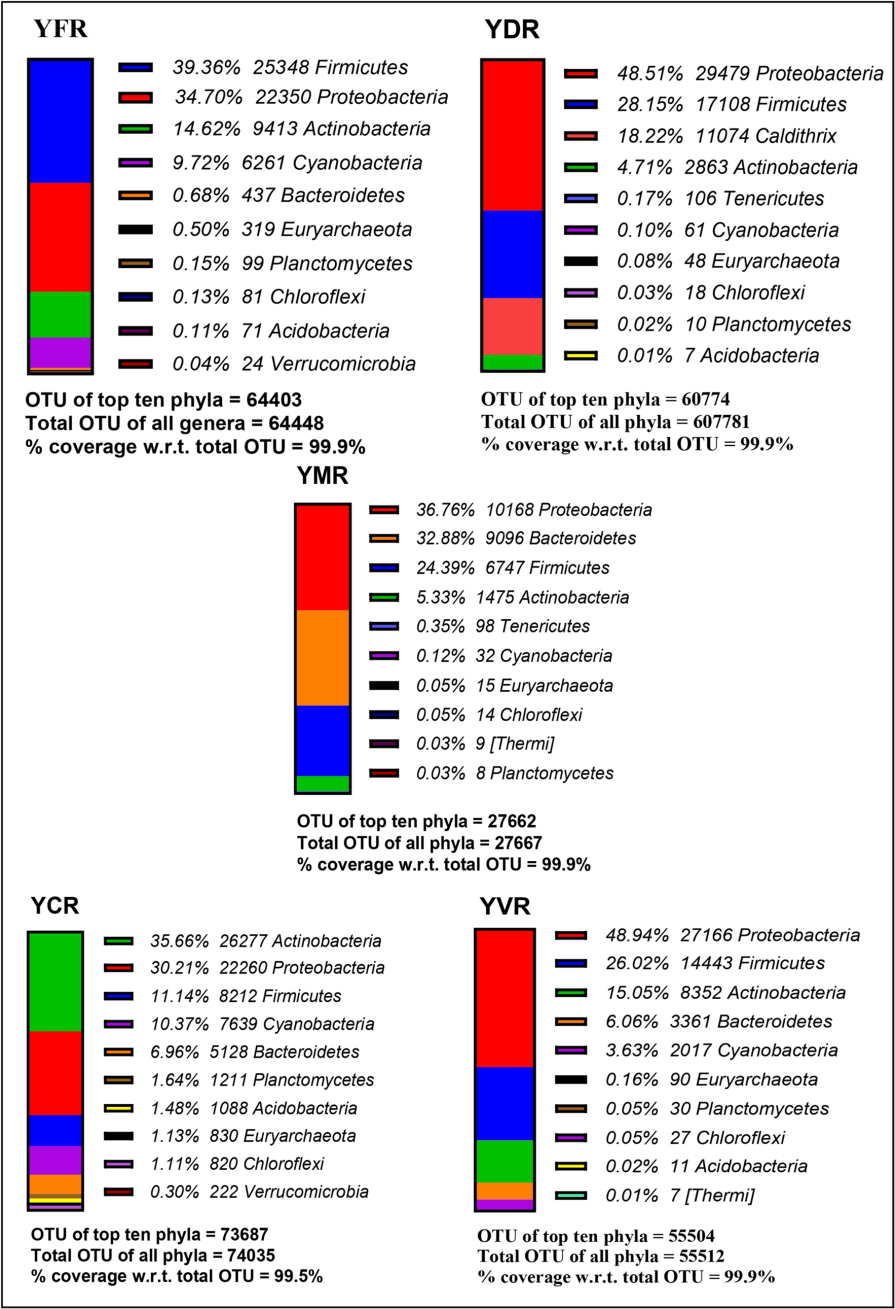
Top ten phyla in the produce of river flood plains soil: this figure shows total OTU of top 10 phyla for each sample and % coverage of these top ten phyla with respect to the total OTU of each sample (YFR, soil A fertilizer produce; YDR, soil A leaf waste compost produce; YMR, soil A municipal waste compost produce; YCR, soil A cow dung manure produce; YVR, soil A vermicompost produce)

**Fig. 4:**
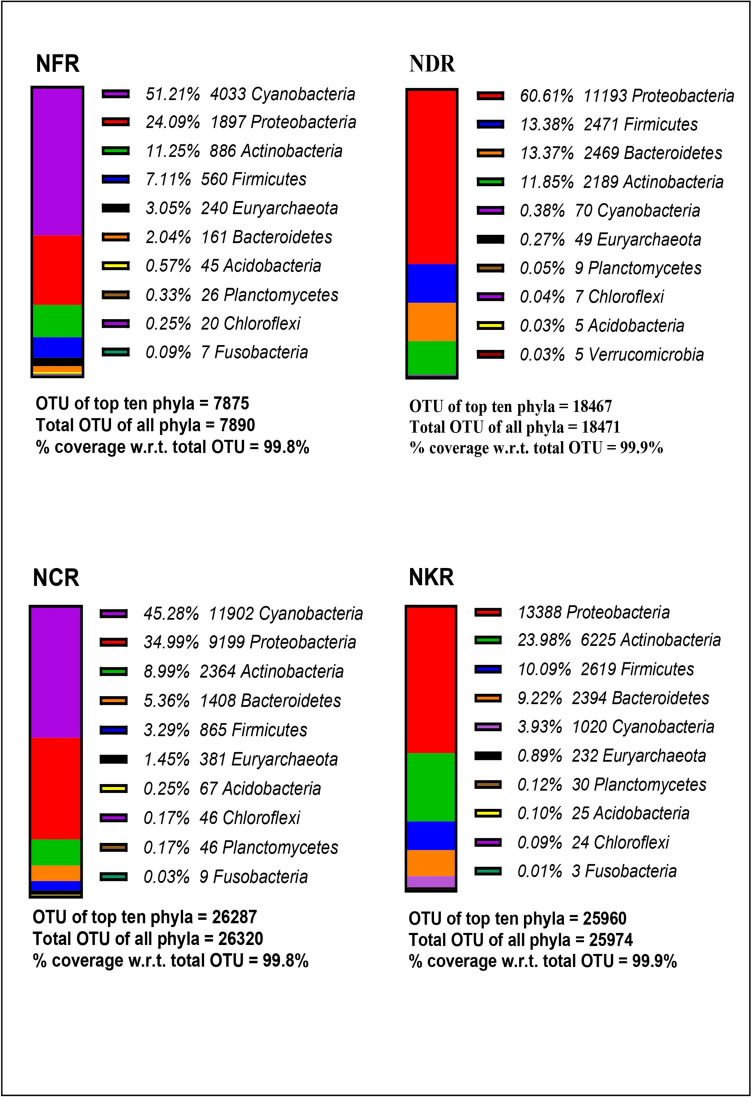
Top 10 phyla in the produce of residential soil: this figure shows total OTU of top 10 phyla for each sample and % coverage of these top ten phyla with respect to the total OTU of each sample (NFRL1, soil B fertilizer produce; NDRL, soil B leaf waste compost produce; NCRL0, soil B cow dung compost; NKRL, soil B kitchen waste compost produce)

In total, 42 phyla were reported across all the produces. The top 10 phyla, which were found to cover almost 99.5–99.9% of the total identified phyla are presented in Figs. 3 and 4. Among all the produce, the common phyla that constitute maximum percentage of the top ten phyla included *Proteobacteria*, *Firmicutes*, *Actinobacteria*, *Cyanobacteria*, and *Bacteroidetes*. *Proteobacteria* formed the maximum abundance in leaf waste compost produce from soil A and followed by vermicompost produce. It was found to be lowest in the fertilizer produce of soil B. *Firmicutes* had the maximum abundance in fertilizer produce from soil A but the fertilizer produces from soil B had the lowest proportion. *Actinobacteria* was the major abundant phyla in cow dung manure produce grown in soil A. It was lowest in proportion in the fertilizer produce of soil B. *Cyanobacteria* was the most abundant phyla in cow dung manure produce grown in soil B. Its lowest proportion was observed in the municipal waste compost produce in soil A. *Bacteroidetes* was the major abundant phyla in the municipal waste compost produce grown in soil A, but the leaf waste compost produces from soil A had the lowest proportion. Few phyla like the *Euryarchaeota*, *Acidobacteria*, *Planctomycetes*, *Verrucomicrobia*, and *Chloroflexi* were present exclusively in abundance in cow dung manure produce of soil A. *Tenericutes* phyla were abundant in leaf waste compost produce grown in soil A. *Fusobacteria and Thermi* were the least abundant phyla (Figs. 3 and 4).

#### Genus’ level

At the genus level, 156 genera were detected, with only a tiny fraction (28 genera) constituting the core microbiome. The core microbiome refers to the set of taxa detected in a high fraction of the population above a given abundance threshold. The count data is transformed to the compositional (relative) abundance to perform such analysis. At a detection threshold of 0.010, the relative abundant genera that constituted the core microbiome included the *Shingobacterium*, *Pseudomonas*, *Achromobacter*, and *Paenibacillus* with a prevalence of 0.6–1.0 at the lowest detection threshold of 0.010–0.125*.* The genera such as *Bacillus, Olivibacter*, *Haloferax*, *Prevotella*, *Streptomyces*, *Cellvibrio*, *Alkaliphilus*, and *Staphylococcus* formed the second abundant group of genera with the prevalence of 0.4 detected at 0.010–0.082 of detection threshold. The genera *Acinetobacter*, *Stenotrophomonas*, *Chryseobacterium*, *Coprococcus*, *Saccharopoluspora*, *Pediococcus*, *Coreynebacterium*, *Streptococcus*, *Rothia*, *Lactobacillus*, *Devosia*, *Cupriavidus*, *Sporosarcina*, *Planomicrobium*, *Arthrobacter*, and *Nocardioides* were present at the lowest prevalence of 0.0–0.2 at detection threshold of 0.010–0.440. The data was visualized with a sample prevalence of 20% and a relative abundance of 0.1% (Supplementary Fig. S1).

The heat map (Supplementary Fig. S2) was constructed at genus taxonomic level with the detailed view mode of < 1500 features. The samples are clustered using the Ward cluster algorithm based on the Euclidean distance measure. The heat map displayed the generic diversity among different produces, such as the YFR had a unique genera representation that included *Corynebacterium*, *Salmonella*, *Brachybacterium*, and *Sporosarcina*. The genera like *Clostridium*, *Brevundimonas*, and *Ochrobactrum* were exclusive to the YDR. The YMR had *Lysobacter*, *Lysinibacillus*, *Flavobacterium*, *Sphingobacterrium*, and *Paracoccus* as unique genera. *Arthrobacter*, *Enterobacter*, *Acinetobacter*, and *Bacillus* were unique to the YVR. The genera like *Saccharopolyspora*, *Truepera*, *Balneimonas*, *Prauserella*, *Gemmata*, *Luteimonas*, *Mycobacterium*, *Nitrospira*, *Alicyclobacillus*, *Flavisolibacter*, *Saccharomonospora*, and *Brevibacillus* were exclusive to the YCR. The *Halococcus*, *Leptotrichia*, *Streptococcus*, *Lactobacillus*, *Neisseria*, *Leuconostoc*, *Saccharothrix*, *Bifidobacterium*, *Rothia*, *Georgenia*, *Rhodococcus*, *Prevotella*, *Exeguobacterium*, *Dialister*, *Gluconobacter*, *Catenibacterium*, *Prevotella-1*, *Bacteroides*, *Haloferax*, *Pediococcus*, *Acetobacter*, *Actinomyces*, *Streptomyces*, *Fusobacterium,* and *Haloarcula* were unique to the NFRL1. The genera like the *Kocuria*, *Steotrophomonas*, *Coprococcus*, *Novispirillum*, *Azospirillum*, *Cupriavidus*, and *Olivibacter* were unique to the NDR. The *Plantomyces*, *Chryseobactrium*, *Amycolatopsis*, *Faecalibacterium*, *Nocardia*, *Halogeometricum*, and *Nocardiodes* were exclusively present in the NKRL.

**Fig. 5:**
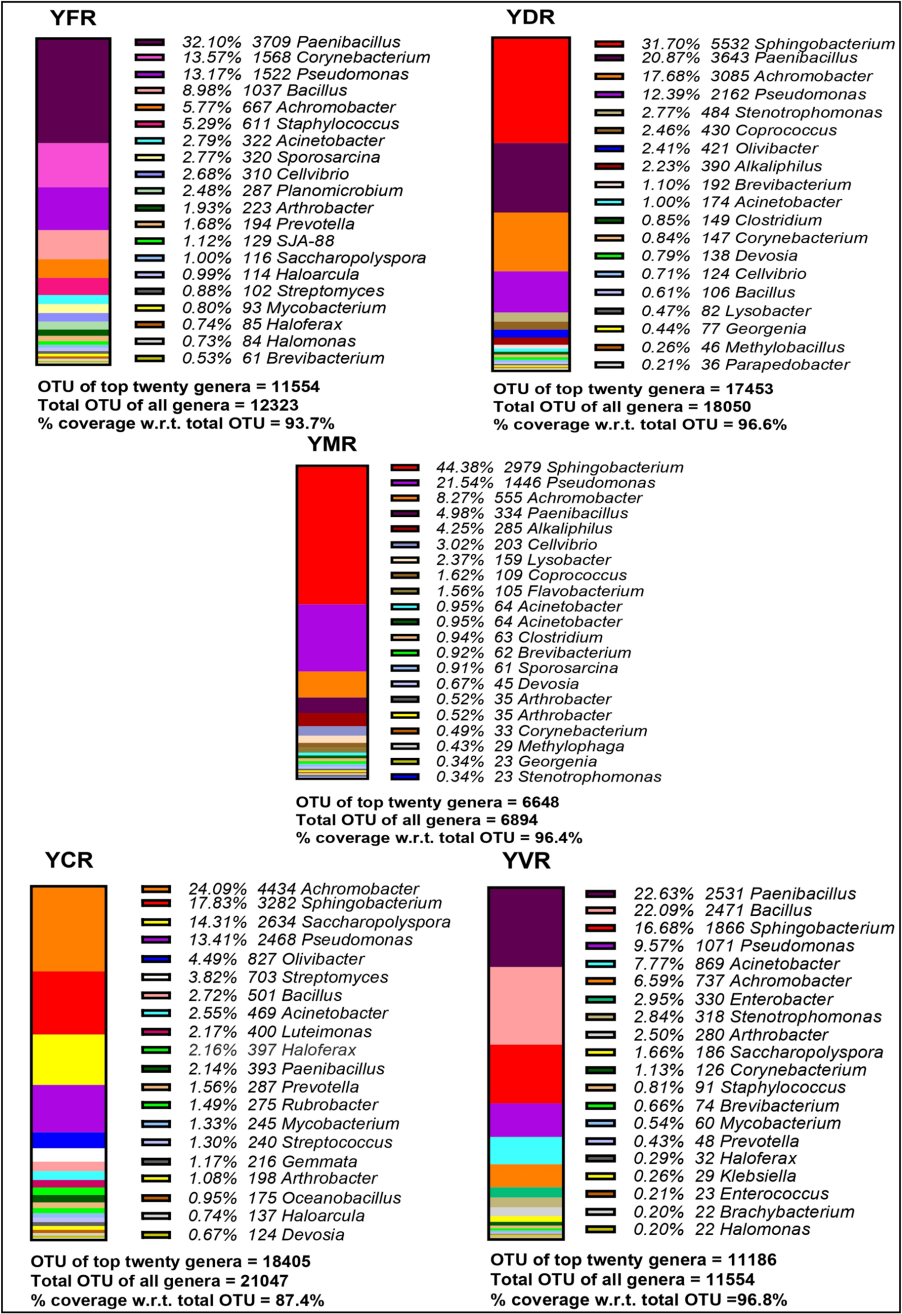
Top 20 genera in the produce of river flood plains soil: this figure shows total OTU of top 20 genera for each sample and % coverage of these top 20 genera with respect to the total OTU of each sample (YFR, soil A fertilizer produce; YDR, soil A leaf waste compost produce; YMR, soil A municipal waste compost produce; YCR, soil A cow dung manure produce; YVR, soil A vermicompost produce)

**Fig. 6:**
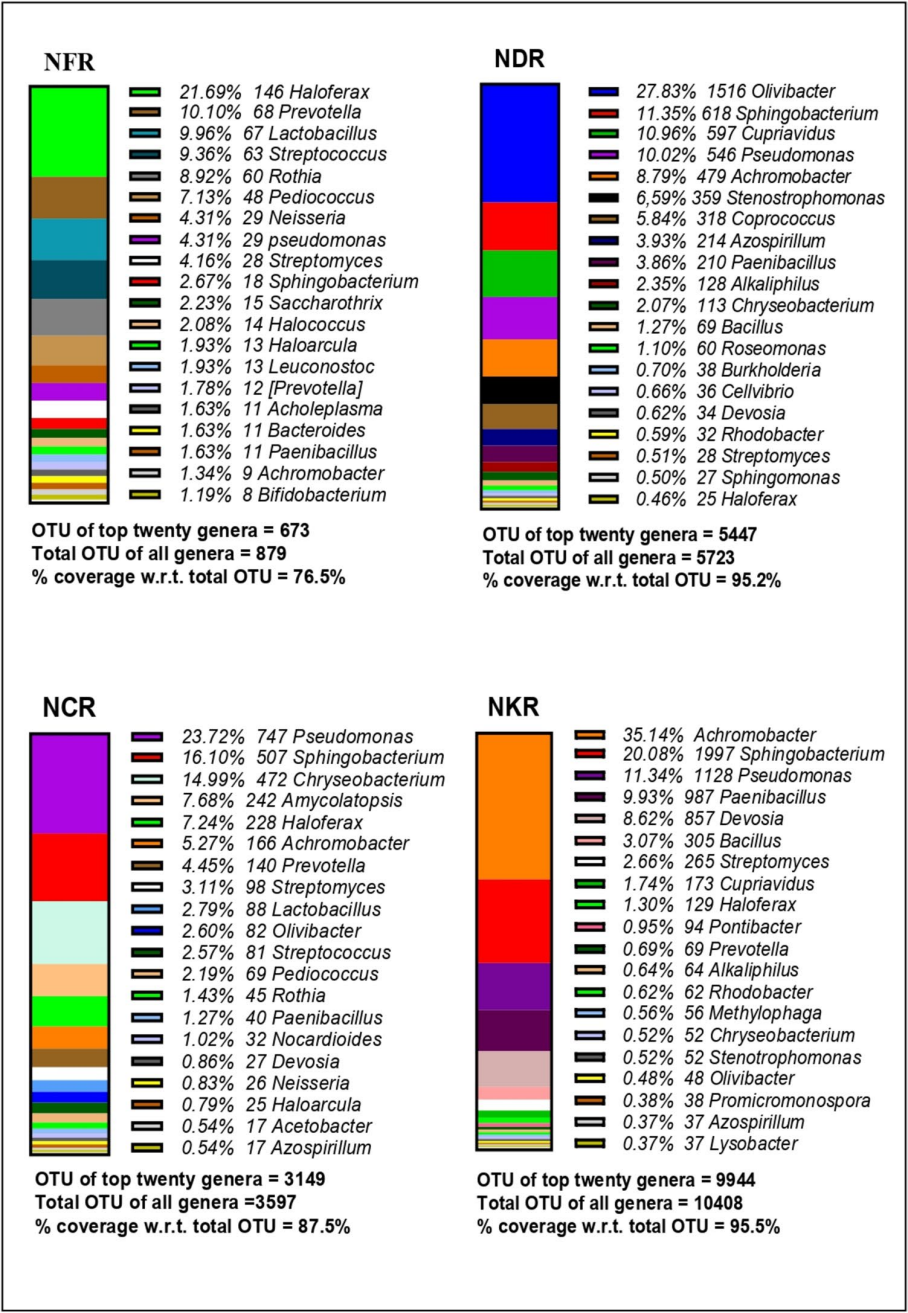
Top 20 genera in the produce of residential soil: this figure shows total OTU of top 20 genera for each sample and % coverage of these top 20 genera with respect to the total OTU of each sample (NFRL1, soil B fertilizer produce; NDRL, soil B leaf waste compost produce; NCRL0, soil B cow dung compost; NKRL, soil B kitchen waste compost produce)

Out of the 156 identified genera, the top 20 genera that covered 76.5–99.9% of the total identified genera were presented in Figs. 5 and 6. Among all the produce, beneficial genera like the *Sphingobacterium*, *Pseudomonas*, *Achromobacter*, *Paenibacillus*, and *Bacillus* were common and constituted the maximum percentage abundance of the top 10 genera. *Sphingobacterium* constituted the maximum abundance in YDR and YMR, while the fertilizer produce of soil B had the lowest proportion of these genera. *Pseudomonas* was the major abundant genera in YCR and NKRL, whereas NFRL1 had its lowest proportion. *Achromobacter* was the major abundant genera in YCR and NKR, whereas NFRL1 had its lowest proportion. *Paenibacillus* constituted the maximum abundance in YDR and YMR, whereas NFRL1 had its lowest proportion. *Bacillus* constituted the maximum abundance in YDR and YMR, whereas NFRL1 had its lowest proportion. Thus, it was seen that the conventional produce had least amount of these beneficial genera **(**Table 2). The pathogenic genera like *Corynebacterium*, *Acinetobacter*, *Cellvibrio*, *Chryseobacterium*, *Enterobacter*, *Streptococcus*, and *Streptomyces* were comparatively abundant in YFR, YVR, YFR, NCRL0, YVR, YCR, and YCR respectively **(**Table 3).

**Table 2:** Beneficial bacterial genus identified in all produces with its corresponding OTUs: this table shows the top ten identified beneficial genera

Genus	Function	YFR	YDR	YMR	YCR	YVR	NFRL1	NDRL	NCRL0	NKRL
*Achromobacter*	phosphate solubilizing,	667	305	559	4434	737	9	479	166	3494
*Alkaliphilus*	halophile	1	390	285	2	1	0	128	1	64
*Arthrobacter*	plant growth promoter	223	31	35	198	280	0	0	0	0
*Azospirillum*	plant growth promoter	0	1	0	3	0	0	214	17	37
*Bacillus*	biopesticide, probiotic, produce antimicrobial peptides	1037	106	20	501	2471	7	69	12	305
*Coprococcus*	plant growth promoters	0	430	109	2	0	0	318	6	2
*Devosia*	plant growth promoter, Nitrogen fixing	3	138	45	124	20	6	34	27	857
*Flavobacterium*	nylon degrading	2	14	105	6	6	4	6	5	3
*Haloferax*	halophilic, anti-fungal and anti-bacterial	85	32	2	397	32	146	25	228	129
*Lactobacillus*	probiotic, decomposer	47	7	2	82	19	67	11	88	27
*Luteimonas*	plant growth promoter	0	11	3	400	1	1	3	0	4
*Lysobacter*	degrades plant pathogenic cell wall	0	82	159	8	2	0	0	0	37
*Leuconostoc*	plant growth promoter, fermentative properties	19	2	0	19	5	13	0	13	5
*Oceanobacillus*	halophile	46	35	13	175	11	1	0	2	0
*Olivibacter*	Decomposer	2	421	1	827	4	2	1516	82	48
*Paenibacillus*	plant growth promoters, nitrogen fixation, bioinsecticide	3709	3643	334	393	2531	11	210	40	987
*Pediococcus*	probiotic	31	4	0	48	8	48	10	69	32
*Planomicrobium*	hydrocarbon chain degrading	287	0	0	1	5	7	0	0	0
*Pontibacter*	plant growth promoter	0	23	18	2	0	2	0	3	94
*Prevotella*	carbohydrate breakdown	194	16	2	287	48	68	15	140	69
*Pseudomonas*	plant growth promoters, nitrogen fixation	1522	2162	1446	2468	1071	29	546	747	1128
*Rothia*	Nitrogen fixer, inhibitory against pathogenic bacteria and fungi	5	2	0	40	4	60	7	45	22
*Rhodobacter*	photosynthetic bacteria	1	2	1	3	1	0	32	8	62
*Saccharopolyspora*	biopesticide	116	25	4	2634	186	0	0	2	0
*Sphingobacterium*	plant growth promoters	129	5532	2979	3282	1866	18	618	507	1997
*Sporosarcina*	plant growth promoter	320	0	61	4	6	0	0	0	0
*Stenotrophomonas*	plant growth promoters	2	484	23	4	318	1	359	7	52

The *Achromobacter*, *Bacillus*, *Paenibacillus*, *Pseudomonas*, and *Sphingobacterium* constituted maximum abundance percentage amongst all the produces (YFR, soil A fertilizer produce; YDR, soil A leaf waste compost produce; YMR, soil A municipal waste compost produce; YCR, soil A cow dung manure produce; YVR, soil A vermicompost produce; NFRL1, soil B fertilizer produce; NDRL, soil B leaf waste compost produce; NCRL0, soil B cow dung compost; NKRL, soil B kitchen waste compost produce)

**Table 3:** Pathogenic genera present in all organic produces with its corresponding OTUs: this table shows the abundant identified pathogenic genera

Genus	Function	YFR	YDR	YMR	YCR	YVR	NFRL1	NDRL	NCRL0	NKRL
*Corynebacterium*	opportunistic pathogen	1568	147	35	69	126	6	2	8	4
*Acinetobacter*	human pathogen	322	174	64	469	869	3	0	11	5
*Brachybacterium*	human pathogenic	32	21	5	15	22	0	2	0	0
*Cellvibrio*	plant pathogen	310	124	203	1	2	0	36	6	18
*Chryseobacterium*	human pathogenic	1	4	0	0	0	8	113	472	52
*Cupriavidus*	opportunistic pathogen	1	4	2	4	0	2	597	14	173
*Enterobacter*	human pathogenic	1	9	5	15	330	0	0	3	1
*Georgenia*	plant pathogen	2	77	23	5	0	5	1	0	13
*Staphylococcus*	human pathogenic	611	18	6	90	91	8	1	10	0
*Streptococcus*	human pathogen	35	3	4	240	10	63	7	81	26
*Streptomyces*	human pathogenic	102	7	0	703	9	28	28	98	265

The genera such as the *Corynebacterium*, *Acinetobacter*, *Cellvibrio*, *Chryseobacterium*, *Streptococcus*, and *Streptomyces* were commonly abundant in all the produces (YFR, soil A fertilizer produce; YDR, soil A leaf waste compost produce; YMR, soil A municipal waste compost produce; YCR, soil A cow dung manure produce; YVR, soil A vermicompost produce; NFRL1, soil B fertilizer produce; NDRL, soil B leaf waste compost produce; NCRL0, soil B cow dung compost; NKRL, soil B kitchen waste compost produce)

#### Species level

The detection resolution was low at the species level and only 108 species were detected. The top 10 abundant species covered 92.5–99.8% of the detected species (Supplementary Figs. S3 and S4). The species like the *hirsuta*, *copri*, and *clausii* constituted the major proportion of the beneficial species present in most of the produces. The *hirsuta spp.* was detected to be abundance in the YCR, while it was completely absent in the NFRL1, NDRL, and NKRL. The *copri* spp. was abundant in the YFR while absent in the YCR. The *clausii* spp. was found abundant in NKRL, whereas absent in the YMR. Interestingly some species, viz., *stercorea*, *sphaeroids*, and *transvalensis*, were common among the soil B compost produces, while they were generally absent in the soil A produces and in the soil B fertilizer produce. Few halotolerant species like the *oncorhynchi* and *rugosa* were present in soil A produce while absent in soil B produce.

The pathogenic species that were commonly abundant included *stutzeri*, *mizutaii*, and *multivorum.* The *stutzeri* spp. was found to have major proportion in the YDR, whereas the least abundance in the NCRL0. The *mizutaii* spp. was abundant in YMR, whereas least abundant in YCR. The *multivorum* spp. was abundant in NDRL, whereas least abundant was in the YFR.

### Bacterial genera comparison

The microbiome detected at the genus level was analyzed and compared. Based on this assorted data, a Venn diagram which clearly showed that the produce of two different soil types had less bacterial genera that are unique to them, such as soil A produces had only 13.9% unique genera, whereas soil B had only 19.8% unique genera. About 43.1% of the bacterial genera were common across all the produces (Supplementary Fig. S5; Supplementary Table S1).

Analyzing the abundance of beneficial and pathogenic genera present across all the produces, it was observed that the YDR had comparatively maximum beneficial microbial genera. In contrast the NFR had the lowest beneficial microbial genera. The NCR was observed to have relatively maximum pathogenic microbial genera, whereas the YDR had the lowest pathogenic microbial genera. The ratio of the beneficial to the pathogenic genera was in the order YDR > NDRL > NKRL > YMR > YCR > YFR > YVR > NFRL1 > NCRL0 **(**Table 4).

**Table 4:** Comparative analysis of beneficial and pathogenic genera OTU: this table presents the percentage and ratio of beneficial to pathogenic genera in different organic and fertilizer produceYFR, soil A fertilizer produce; YDR, soil A leaf waste compost produce; YMR, soil A municipal waste compost produce; YCR, soil A cow dung manure produce; YVR, soil A vermicompost produce; NFRL1, soil B fertilizer produce; NDRL, soil B leaf waste compost produce; NCRL0, soil B cow dung compost; NKRL, soil B kitchen waste compost produce

	YFR	YDR	YMR	YCR	YVR	NFRL1	NDRL	NCRL0	NKRL
Total OTU at the genus level	12,323	18,050	6894	21,047	11,554	879	5723	3597	10,408
Total OTU of beneficial genera	9059	16,696	6208	16,434	9724	508	4601	2225	9455
Percentage of beneficial genera	**73.5%**	**93%**	**90%**	**78%**	**84.2%**	**57.8%**	**80.4%**	**61.9%**	**90.8%**
Total OTU of pathogenic genera	2984	588	3471	1611	1459	123	787	703	557
Percentage of pathogenic genera	**24%**	**3%**	**5%**	**8%**	**13%**	**14%**	**14%**	**20%**	**5%**
Ratio of beneficial and pathogenic genera	**3.04**	**28.4**	**17.9**	**10.3**	**6.6**	**4.4**	**5.8**	**3.2**	**16.9**

## Discussion

The present study is the first of its kind to study the organic and conventional produce using 16S RNA microbiome profiling metagenomics. 16S RNA profiling has helped us to analyze the microbial richness and diversity, including beneficial and pathogenic genera, of a leafy vegetable grown organically. Based on the percentage abundance of the beneficial and the pathogenic genera, a comparison was made to highlight the ratio of beneficial to pathogenic genera among the produces of popular farming practices. This is great progress from the earlier studies in which the limited observation of few microbes like mesophilic aerobic bacteria, *E. coli*, and coliform, using the traditional microbiological culture techniques, was popularly done in organic cultivar (Becker et al. 2019; Maffei et al. 2013; Merlini et al. 2018; Shafie 2021). Moreover, this study has used different composts as well different kinds of soil to observe the impact of composts and soils on the microbial richness and diversity of the organic cultivars.

The broad range of the OTU means that the microbial richness varied among produces grown using different organic composts, and it also varied with the soil variation. Soil A produce had more microbial richness, while soil B taken from the garden area had a much lesser microbial count as the origin of soil A was from the riverbank. Furthermore, the addition of compost increased the microbial richness even more in most of the organic produces of both soils compared to the conventional produce.

As far as the alpha diversity is concerned, the observation made based on refraction curve depicted that the produces from the soil type had similar alpha diversity conferring that variation in the soil had not much effect on species richness. However, alpha diversity was affected by the type of compost used as the cow dung manure produce and the fertilizer produce had the higher alpha diversity than the urban organic waste compost produce, like leaf waste compost produce, kitchen waste compost produce, and municipal waste compost produce. This could be due to the nutrient richness of cow dung manure which supports microbial diversity and richness.

The dendrogram prepared based on beta diversity results is the assessment of the intergroup microbiome comparison. Here we see that the urban organic waste composts, such as the leaf waste compost produce, municipal waste compost produce, and kitchen waste compost produce were placed closely in the dendrogram. The placement of the groups together in the dendrogram indicates the origin similarity of the constituent microbiome and their ancestral homogeneity.

The OTU assessment at different taxonomic levels showed a total of 42 phyla, 106 classes, 180 orders, 186 families, 156 genera and 106 species. The metagenomic study at the phylum level showed that the cow dung manure produce in both soil types had the highest number of OTU. The major abundant phyla across all the produce included *Cyanobacteria*, *Proteobacteria*, *Actinobacteria*, *Firmicutes*, and *Bacteroidetes*. The cow dung manure produce grown in soil B had the maximum abundance of *Cyanobacteria* which is a nitrogen fixer that helps in the plant growth (Percival and Williams 2013). In humans, it has been found to act as an opportunistic pathogen which may cause gastroenteritis in immunocompromised patients (Apeldoorn et al. 2007). *Proteobacteria* has a similar function and was found abundant in leaf waste compost produce of soil A (Rizzatti et al. 2017). *Actinobacteria* was highest in cow dung manure produce in soil A. It is a decomposer for all types of organic compounds that helps in recycling of the nutrients and also acts as a biocontrol against soil-borne plant pathogens (Sharma and Salwan 2018); and also is opportunistic pathogen to humans. It may be associated with tuberculosis and also may cause skin and soft tissue infections in immunocompromised patients (Sowani et al. 2017). *Firmicutes* had the maximum abundance of in the fertilizer produce in soil A. *Bacteroidetes* had the maximum abundance in the municipal waste produce. Firmicutes and Bacteroidetes are probiotics and they are polysaccharide degraders in the gut like methanogens. But the latter may also act as an opportunistic pathogen in humans (Crouch et al. 2022).

Another observation of interest in the present study was that adding of compost increased the beneficial genera in all the compost produce irrespective of the soil type compared to conventional produce. At the genus level, the beneficial microbiomes that form a major part of the OTU in all the produce were the *Achromobacter*, *Bacillus*, *Paenibacillus*, *Pseudomonas*, and *Sphingobacterium.* The *Achromobacter* are phosphate-solubilizing genera that help recycle the phosphorus mineral, which is a macronutrient essential for the growth of the plant (Isler et al. 2020). These were found to be abundant in cow dung manure produce grown in soil A. *Bacillus* genera were found in abundance in cow dung and vermicompost produce. They secrete vast variety of antimicrobial peptides (AMPs) that have been shown to have a broad spectrum of activity against pathogenic microbes (Lee et al. 2019; Crawford and Daum 2008). In the case of plants, they may enhance stress tolerance and adaptation to climate change (Hashem et al. 2019). The *Paenibacillus* genera, abundant in the fertilizer produce from soil A, is a plant growth promoter and they help in fix atmospheric nitrogen (Grady et al. 2016). The genera *Pseudomonas*, abundant in cow dung manure produce of soil A, are root colonizers that help in nitrogen fixation and compete with plant pathogens for essential nutrients (Das et al. 2020). They often produce secondary metabolites like siderophores and organic acids for better nutrient accusation (Sah et al. 2017). The genera *Sphingobacterium* abundant in leaf waste compost produce of soil A are rhizobacteria that aid in plant growth (Ahmed et al. 2014).

At the species level, *hirsute*, *copri*, and *clausii* were detected as beneficial species. They were at comparatively higher abundance in cow dung manure produce, fertilizer produce, and kitchen waste compost produce, respectively. The *hirsuta* species is a methanotroph that act as a decomposer and also as a rhizobacterium that aid in nitrogen fixation (Chen et al. 2022). Species *clausii* have probiotic potential comparable to the lactic acid bacteria. They play a vital role in directly maintaining gastrointestinal microbial balance, in particular, and increasing the entire body’s immunity, in general (Ghelardi et al. 2022). The *copri* species are generally found abundant in the human gut as these are the probiotic species that help metabolize fiber and hemicellulose degradation (Yeoh et al. 2022).

The addition of compost decreased the pathogenic genera in all the compost produces in soil A as well as in soil B compared to the conventional produce. The organic compost produce had comparatively lower pathogenic microbiome irrespective of the soil types. The pathogenic bacterial genera detected in high abundance included *Corynebacterium*, *Acinetobacter*, *Cellvibrio*, *Chryseobacterium*, *Streptococcus*, and *Streptomyces*. The *Corynebacterium* genera are known to be pathogenic to plants, animals, and humans. In plants a few species of this genera cause vascular disease that spreads through the vascular system, disrupting the water-conducting vessels leading to wilts, while others cause hypertrophic diseases, which is the uncontrolled proliferation of meristematic tissues (Lelliott 2011). They cause a human skin disorder called pitted keratolysis (Nicolas et al. 2010). Some strains of this genera may produce exotoxins causing diptheria (Smith and Oram 2009). The *Acinetobacter* genera, found abundant in vermicompost produce, can colonize many body surfaces and cause infection in almost any organ system. The most common infections are respiratory (pneumonia), bloodstream (bacteremia), urinary tract, wound, skin and soft tissue, and burn infections; osteomyelitis secondary to trauma; and meningitis (Tiwari et al. 2015). The genus *Cellvibrio*, present in highest amount in the municipal waste compost produce, has been reported as degraders of cellulose, dextran, xylan, chitin, and starch, which constitute the plant woody layer and are potential plant pathogens (Suarez et al. 2014). The *Chryseobacterium*, abundant in cow dung manure produce of soil B, genera are associated with nosocomial infections in neonates and immunocompromised patients. Some species are associated with urinary tract infections and meningitis (Rai et al. 2012). The *Streptomyces* genera, abundant in cow dung manure produce from soil A, are a common cause of *actinomycetoma* or *mycetoma*, a chronic suppurative infection of the skin and underlying soft tissue and bone; pulmonary allergies; and also pneumonia in some cases (Sharma et al. 2014). *Staphylococcus*, abundant in cow dung manure produce by soil A, causes skin abscesses that are warm, painful, collections of pus below the skin surface, and *Staphylococcus cellulitis* is a spreading infection that develops under the skin producing pain and redness (Kobayashi et al. 2015).

The identified pathogenic species in all the produce included *stutzeri*, *mizutaii*, and *multivorum*. The *stutzeri*, found higher in leaf waste compost produce of soil A, has been associated with osteomyelitis, arthritis, bacteremia, endocarditis, endophthalmitis, pneumonia, empyema, urinary tract infections, and meningitis in patients suffering from chronic liver and renal diseases, or immunosuppression. Infections in otherwise healthy patients have also been reported, such as brain abscesses, pneumonia, empyema, and vertebral osteomyelitis (Lalucat et al. 2006). The *mizutaii* species, reported highest in municipal waste compost produce, have been found to degrade antibiotic sulfamethoxazole which is used to control aquatic animal diseases. These residues are hard to eliminate and thus may accumulate at higher trophic levels by getting into the food chain, becoming a potent threat to human health (Song et al. 2021). The *multivorum* species, detected highest level in leaf waste compost produce of soil A, have been associated with peritonitis, septicemia, bacteremia, chronic respiratory infection in patients with immunosuppression, and colonization of the air passage in patients with cystic fibrosis (Pernas-Pardavila et al. 2019). These have been mostly isolated from the blood and urine specimens of humans and are found to be resistant to antimicrobial agents such as aminoglycosides, quinoles, trimethoprim-sulphamethoxazole, and beta-lactam (Barahona and Slim 2015).

The addition of compost improved the beneficial-to-pathogenic ratio in both soil types. This means the addition of compost increases the benefits and decreases the pathogenic genera. Leaf waste compost has shown a maximum increase in the beneficial-to-pathogenic ratio, indicating leaf waste compost is a rich biofertilizer. Kitchen waste compost produce and municipal waste compost produce have also shown increase in beneficial-to-pathogenic ratio but lower than leaf waste compost. Interestingly, the cow dung manure produce had a much lower beneficial-to-pathogenic genera ratio, indicating that it increases not only the beneficial microbes but also the pathogenic microbes than the urban organic composts such as the leaf waste compost produce, kitchen waste compost produce, and the municipal waste compost produce. The cow dung manure and vermicompost of cow dung should be bioremediated further to enhance their fertilizing potential.

So, our study partially accepts the hypothesis: “Organic produce has higher counts of both the beneficial and pathogenic microbes.” The organic farming produce does have high count of the beneficial microbiome but generally low count of pathogenic microbiome compared to the conventional produce in both the soil types.

The next-generation sequencing has helped us to detect all the 16S rRNA present in the sample. Some of these RNAs were of unknown species and others were assigned a name but their classification and functions were not clear or available in the literature. This limited our study as we could not assign them as the beneficial or pathogenic microbes and include them in our analysis. The other limitation was that the present study was carried out on an experimental scale and thus we recommend for a field-level experiment.

## Supplementary Information

Below is the link to the electronic supplementary material.

Supplementary file1 (PDF 1347 KB)

## Data Availability

All the data generated and analyzed in the study are provided in this manuscript, and its supplementary files.
