# Supplementary material for: 16S rRNA metagenomic profiling of red amaranth grown organically with different composts and soils
Source: Appl Microbiol Biotechnol. 2024 Jan 15;108:129. doi: 10.1007/s00253-023-12982-7 (PMC10789846; doi:10.1007/s00253-023-12982-7)
Supplement: Supplementary file 1 [file 253_2023_12982_MOESM1_ESM.pdf]

16S rRNA metagenomic profiling of red amaranth grown organically with different composts and soil

Pooja Sharma[a], Sophayo Mahongnao[a], Arif Ahamad[b], Radhika Gupta[a], Anita Goel[a], Narendra Kumar[a] and Sarita Nanda[a*]

[a]Department of Biochemistry, Daulat Ram College, University of Delhi, Delhi - 110007, India.

[b]Department of Environmental Science, Jamia Millia Islamia University, New Delhi – 110025, India.

***Correspondence:**

Sarita Nanda
saritananda123@gmail.com
Department of Biochemistry, Daulat Ram College, University of Delhi,
4, Patel Marg, Maurice Nagar, Delhi - 110007, India.

Supplementary figures

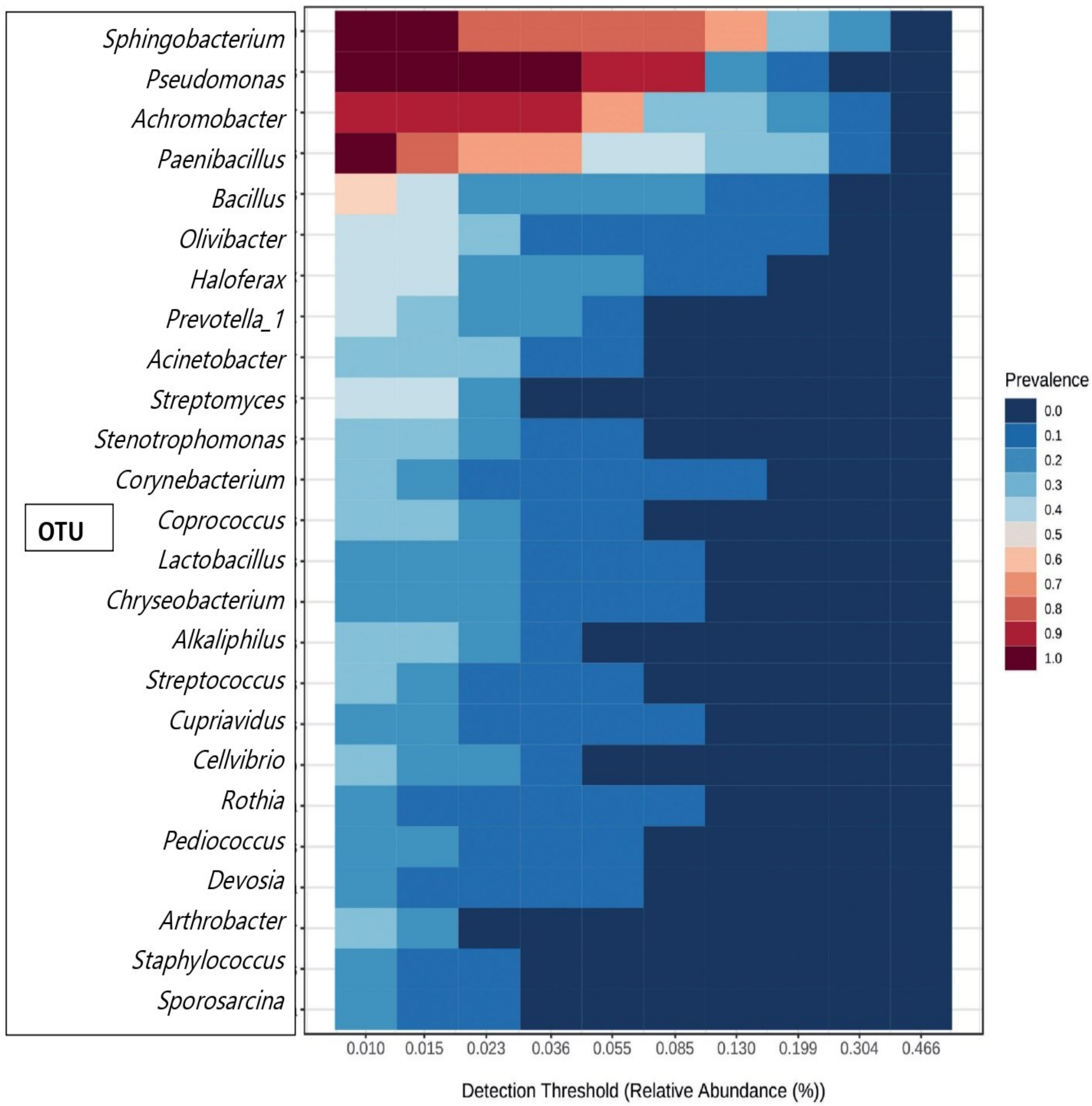

Supplementary Figure S1. Core Microbiome: The core microbiome detected in different produce: The core microbiome is the set of taxa detected in a high fraction of the population above a given abundance threshold. The count data is transformed to compositional (relative) abundance to perform such analysis.

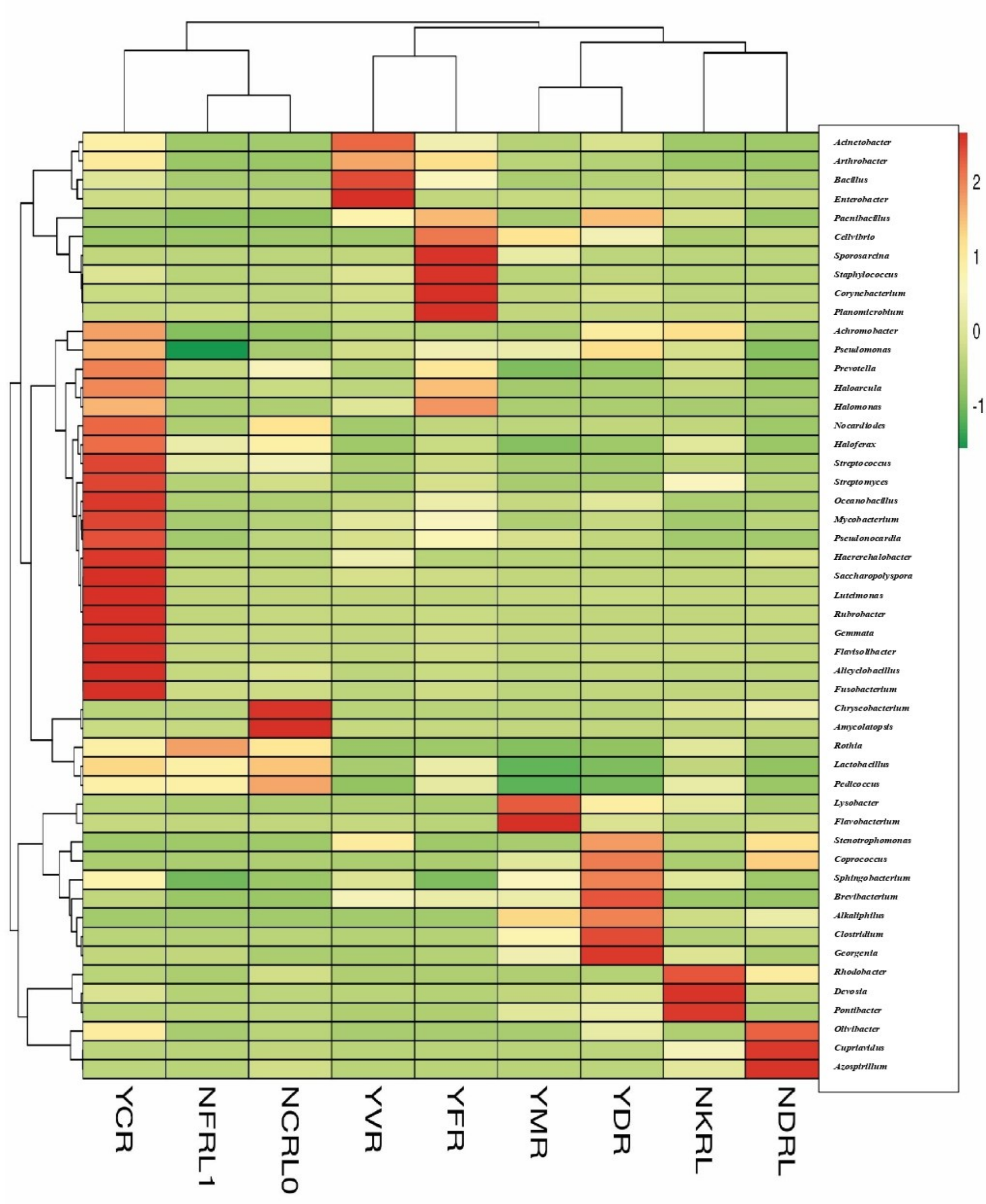

Supplementary Figure S2. Heat Map: The heat map representing the generic diversity among different produce (YFR: Soil A fertilizer produce, YDR: Soil A leaf waste compost produce, YMR: Soil A municipal waste compost produce, YCR: Soil A cow dung manure produce, YVR: Soil A vermicompost produce, NFRL1: Soil B fertilizer produce NDRL: Soil B leaf waste compost produce, NCRL0: Soil B cow dung compost NKRL: Soil B kitchen waste compost produce)

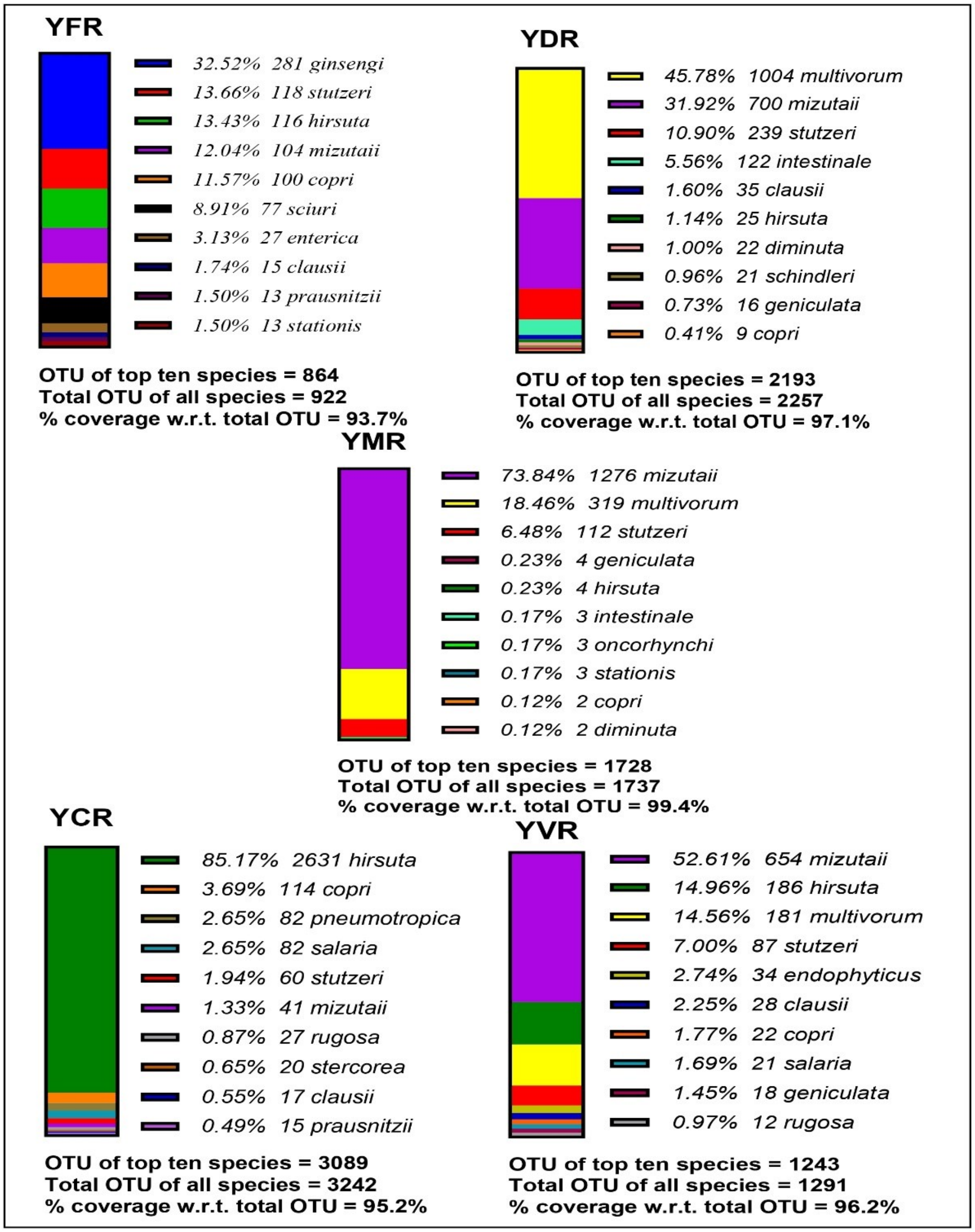

Supplementary Figure S3. Top ten species in the produce of River flood plains soil: This figure shows total OTU of top ten species for each sample and % coverage of these top ten species with respect to the total OTU of each sample (YFR: Soil A fertilizer produce, YDR: Soil A leaf waste compost produce, YMR: Soil A municipal waste compost produce, YCR: Soil A cow dung manure produce, YVR: Soil A vermicompost produce)

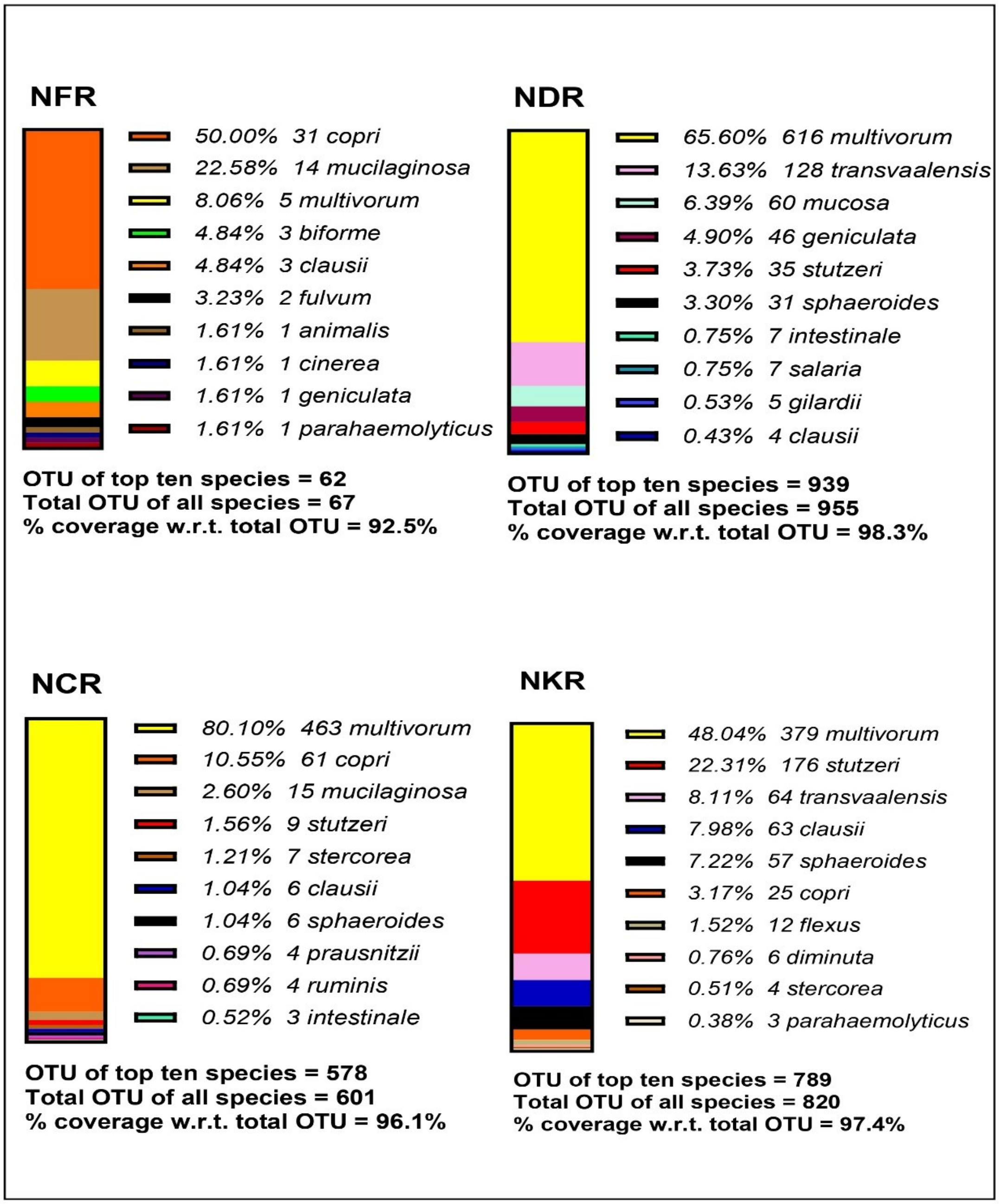

Supplementary Figure S4. Top ten species in the produce of residential soil: This figure shows total OTU of top ten species for each sample and % coverage of these top ten species with respect to the total OTU of each sample (NFRL1: Soil B fertilizer produce NDRL: Soil B leaf waste compost produce, NCRL0: Soil B cow dung compost NKRL: Soil B kitchen waste compost produce)

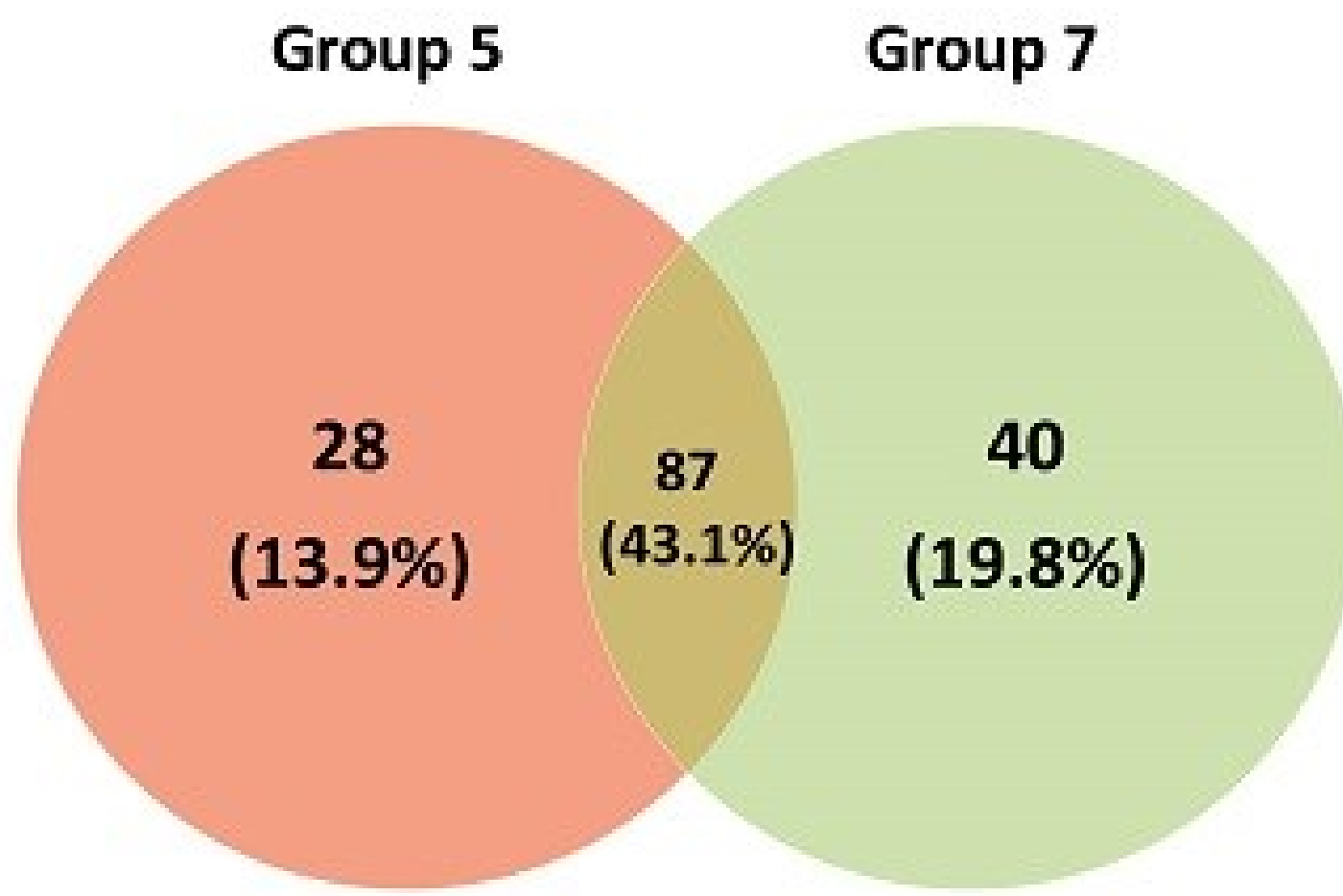

Supplementary Figure S5. Venn Diagram: This Venn diagram compares the overall diversity of bacterial genera across the produces grown in the two soils, soil A (Group 5)and soil B (Group 7)

Supplementary table

Unique genera in Group 5	Unique genera in Group 7	Common genera in Group 5 and 7
Aerococcus	*Actinobacillus*	*Acetobacter*
Aeromicrobium	*Azospirillum*	*Actinomyces*
Ardenscatena	*Bdellovibrio*	*Agromyces*
Arthrobacter	*Burkholderia*	*Alkaliphilus*
Brachybacterium	*Candidatus Portiera*	*Amycolatopsis*
Bradyrhizobium	*Capnocytophaga*	*Coprococcus*
Janibacter	*Fructobacillus*	*Enterobacter*
Lysobacter	*Kineococcus*	*Erwinia*
Parvimonas	*Nocardia*	*Geodermatophilus*
Pigmentiphaga	*Novispirillum*	*Georgenia*
Planifilum	*Opitutus*	*Gluconobacter*
Planomicrobium	*Pimelobacter*	*Glycomyces*
Prauserella	*Polaribacter*	*Granulicatella*
Prosthecobacter	*Pseudochrobactrum*	*Halococcus*
Saccharomonospora	*Roseomonas*	*Halogeometricum*
Saccharopolyspora	*Saccharothrix*	*Halogranum*
Salmonella	*Saccharothrix*	*Halorubrum*
Steroidobacter	*Sedimentibacter*	*Kaistobacter*
Tetrathiobacter	*Sinorhizobium*	*Kocuria*
Truepera	*Turicibacter*	*Leuconostoc*
A17	*Vibrio*	*Luteimonas*
Brevibacterium	*[Eubacterium]*	*Lysinibacillus*
Dietzia	*Bifidobacterium*	*Olivibacter*
Enterococcus	*Candidatus*	*Paracoccus*
Prauseria	*Solibacter*	*Pediococcus*
Rhodoplanes	*Chryseobacterium*	*Promicromonospora*
Sporosarcina	*Collinsella*	*Rhodobacter*
Symbiobacterium	*Deinococcus*	*Skermanella*
	Dyadobacter	*Succinivibrio*
	Gemmata	*Xylanimicrobium*
	Haloterrigena	*[Prevotella]*
	Inquilinus	*Achromobacter*
	Lachnospira	*Acinetobacter*
	Lactococcus	*Agrobacterium*
	Mycoplana	*Alicyclobacillus*
	Neisseria	*Anaerolinea*
	Novosphingobium	*Bacillus*
	Oscillospira	*Bacteroides*
	Porphyromonas	*Balneimonas*
	Sphingobium	*Brevibacillus*
	Sutterella	*Brevundimonas*
	Trueperella	*Catenibacterium*
		Cellvibrio
		Clostridium
		Corynebacterium
		Cupriavidus
		Devosia
		Dialister
		Exiguobacterium
		Faecalibacterium
		Flavisolibacter
		Flavobacterium
		Fusobacterium
		Gordonia

		Haemophilus
		Haloarcula
		Haloferax
		Halomonas
		Klebsiella
		Lactobacillus
		Leptotrichia
		Megasphaera
		Methanobrevibacter
		Methylobacterium
		Microbacterium
		Mycobacterium
		Nocardioides
		Oceanobacillus
		Ochrobactrum
		Paenibacillus
		Phascolarctobacterium
		Planctomyces
		Pontibacter
		Prevotella
		Pseudomonas
		Pseudonocardia
		Rhodococcus
		Rothia
		Rubrobacter
		Ruminococcus
		Sphingobacterium
		Sphingomonas
		Staphylococcus
		Stenotrophomonas
		Streptococcus
		Streptomyces
		Veillonella

Supplementary Table S1. Comparative analysis of the microbiome: This table shows the unique and common microbial genera of soil A and soil B produce (Group 5 and Group 7 respectively). The Venn diagram was created on the basis of this comparative analysis.
